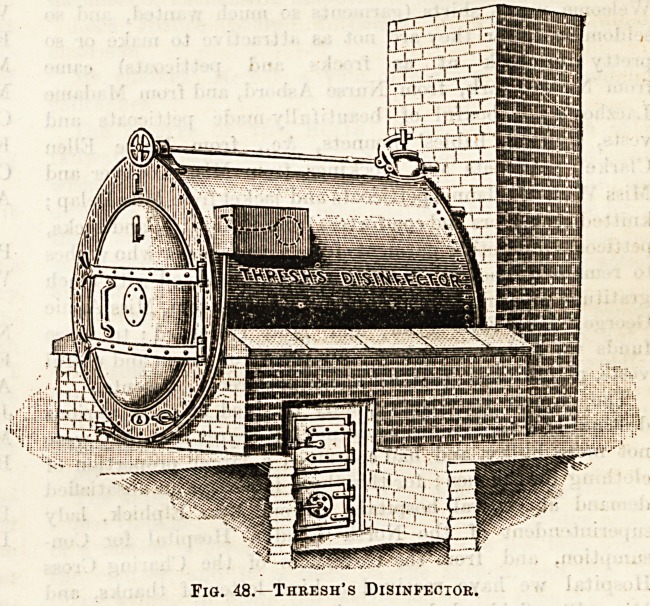# "The Hospital" Nursing Mirror

**Published:** 1897-01-02

**Authors:** 


"The Hospital" Nursing Mirror.

Being the Nursing Section of "The Hospital."

[Contributions for this Section of "The Hospital" should be addressed to the Editor, The Hospital, 28 & 29, Southampton Street, Strand, London, W.C., and should have the word "Nursing" plainly written in left-hand top corner of the envelope.]

### News from the Nursing World.

### ROYAL NATIONAL PENSION FUND FOR NURSES.

The efforts of those nurses, about one-fifth of the whole number, who have shown commendable public spirit and a desire to help each other by collecting on behalf of the Junius S. Morgan Benevolent Fund, have in the last two months resulted in raising in donations and subscriptions a capital sum of several thousand pounds. Many of the nurses who are members of the Pension Fund have spontaneously sent an annual subscription of 1s. and upwards as their own contribution to the Benevolent Fund. These subscriptions amount to nearly £90, and there is a general wish that they should be increased to £100 per annum, so that we shall be glad to receive and acknowledge annual subscriptions from 1s. a year and upwards from any nurse willing to contribute in this way to help members of the Fund who may become disabled from no fault of their own. Only £11 is wanted, and we are confident there must be more than two hundred nurses willing to subscribe 1s. a year with so excellent an object. Policyholders who have not yet subscribed are requested to send in their names at once to the Secretary of the Fund, 28, Finsbury Pavement, London, E.C.

### A NEW CAREER FOR MEDICAL WOMEN.

A remarkable proposal is reported to have been made by a lady member of the Hampstead Guardians at a recent meeting of the Board. Miss Girdlestone, the superintendent of nursing at the infirmary, had sent in her resignation; and, in discussing the question of her successor, Mrs. Finley said she "hoped a lady doctor might be appointed; if that were done they might be able to do without a superintendent of nursing." It would be interesting to know why a training in the profession of medicine should be supposed to carry with it all the qualifications desirable for the head of the nursing department, or how the duties of doctor and nurse could be amalgamated! Fortunately, Mrs. Finley's colleagues on the Board held other views, and the question of filling the vacancy was referred to the Nursing Committee. A resolution was passed accepting Miss Girdlestone's resignation "with regret."

### ROYAL HOSPITAL, RICHMOND.

A Christmas appeal is being issued by the managers of this hospital on behalf of the children's ward, opened by the Duchess of York last year. These extra beds, which are always full, have cast a heavy additional burden on the resources of the hospital. Repairs and the provision of certain necessary surgical equipments for the new ward have led to a deficiency of nearly £200 on the final accounts, and for help to pay off this and for new annual subscriptions the inhabitants of the "Royal borough" are now being asked to give their support. If people who do not know the hospital would take the trouble to pay it a visit, and spend an hour or two in hearing of its needs, the money required would quickly be forthcoming. It is a blot upon the reputation of Richmond and its neighbourhood that its "Royal" hospital should thus need to plead for help.

### NEW HOSPITAL FOR WOMEN, MELBOURNE.

In October a hospital was opened in Melbourne for the special treatment of women by women doctors, an institution which it is proposed by its founders shall ultimately be much extended, and managed on the lines of the New Hospital for Women in London. The medical women of Melbourne have been keenly interested in the formation of the scheme, which at present consists only of an out-patient department, funds not permitting as yet the opening of a ward for in-patients.

### NURSING AT THE CITY OF LONDON UNION INFIRMARY.

It is to be hoped that the Local Government Board will remain firm in requiring a reorganisation of the nursing at this infirmary. The desirability for carrying into effect a better scheme of training was strongly put before the Guardians in a recent letter from the Local Government Board; but at a special meeting of the Board the other day Mr. Lyon put forward the argument that probationers could not be trained upon the cases admitted into the infirmary, in which he was supported by Mr. Fraser Black and others, who contended "that the infirmary was not a hospital, and therefore the practice was not such as would train nurses in their duties." In spite of the counter speeches of Mr. White and Mr. Hadden, a resolution that that portion of the nursing scheme relating to the appointment of probationers should be rescinded was carried by 20 votes to 17.

### NEWS FROM DUBLIN.

Lord Roberts, Commander of the Forces in Ireland, accompanied by Lady and the Hon. Ada Roberts, gave much pleasure to the nurses of the Rotunda Hospital, Dublin, by going to hear the carol singing through the wards on Christmas Eve. A number of guests were invited to meet them, and tea was served in the board-room, where the visitors were received by the master and his sister, Miss Purefoy, and Miss Sara Hampson, the popular superintendent, who is about to retire from the post after seven years' service. Miss Lucy Ramsden, whose appointment will be found in another column, has been elected to succeed her.

### NURSES IN SHANGHAI.

We are glad to learn that the nurses of the Shanghai Municipality have arrived safely at their destination. They received a warm welcome, and must have been highly flattered by the complimentary remarks which appeared in the *Shanghai Times* of November 7th last, which says: "The advent in Shanghai of the three ladies especially engaged by the Municipal Council for private nursing has been looked forward to for some time with great interest by our medicos and the public alike, and it is with the greatest pleasure that we realise that they are here. The pleasure is enhanced twofold on personal acquaintance with these ladies, who approach as near the ideal as we feel they personify the real nurse, and promise all the most fastidious can desire." Nurses Campbell, Gladwell, and Low thus commence their new duties with the best of good wishes, and in these we heartily join.

### THE SUPERANNUATION ACT IN OPERATION.

A SAD example of what may be expected from the effect of the new Poor Law Officers' Superannuation Act is reported from the Bromsgrove Board of Guardians. At a recent meeting it appeared that no application had been received to an advertisement for an assistant nurse, and one Guardian pertinently suggested that a reason might be found in the reduction of salary under the new Act. The Clerk proposed that the difficulty might be met by an equivalent increase in the salary. "But," the Chairman remarked, "in this case they would have to increase the salaries of the master and the other officials also"; and the final regrettable result is one that may be expected all over the country, "It was decided to advertise again, but to omit the condition that applicants must have received training as nurses."

### "LUXURY FUNDS."

A LONDON matron of our acquaintance has an excellent system of keeping certain "purses," known as "luxury funds," which are the means of providing many a little matter not to be brought under the head of ordinary hospital expenditure, but none the less desirable. There is, for example, a "Patients' Luxury Fund," added to now and again by some kind friend wishful to spend a few pounds in charity, from which a small weekly grant to a poor family whose bread-winner has been struck down by illness or accident is an untold boon in helping to ease the patient's mind, and so quicken recovery. And a wheel-chair, a cushion, bed-jackets, and a hundred other little needs are made possible by the careful outlay of this money. Another fund is for the benefit of the nurses, to which members of the hospital committee and other friends interested in their welfare contribute an occasional £5 or £10, and this provides additions to the sitting-room library, a new table cover or easy chair, or possibly a summer expedition somewhere. The plan is a good one, well worthy of imitation.

### A LOSS TO THE ROYAL BRITISH NURSES' ASSOCIATION.

MISS ALICE RAVENHILL'S resignation of the post of Secretary of the Royal British Nurses' Association, on account of ill-health, has been accepted with much regret by the Executive Committee. During Miss Ravenhill's three years of office she has worked with her whole heart for the good of the Association, and by her ready courtesy and tact, no less than by her experience and knowledge of business, has proved herself to be eminently fitted for such a position, with its varied requirements and responsibilities, one by no means easy to fill, and for which it is very difficult to find exactly the right person. Miss Ravenhill has taxed her health to the uttermost in her devotion to her duties as secretary, and even a long rest has not been sufficient to enable her to continue in it through the coming year. By all with and for whom she has worked, from H.R.H. Princess Christian (the president) to the nurse-members of the Association, Miss Ravenhill's loss will be much felt; her influence has been always on the side of a wide-minded and fair policy, and the nurses have found her a real friend. We sincerely hope that a further complete rest and change will quite restore her to health and strength.

### MISLEADING ASSERTIONS.

ON December 17th an application was made in the case of Breay v. Browne for leave to appeal from the decision of the Divisional Court which had, in the words of Mr. Odger, counsel for the plaintiff, "set aside the verdict of the jury and entered judgment for the defendant, whereas judgment had been given for the plaintiff in the court below." An account of the proceedings in this case we publish on page 121. With extraordinary effrontery, a journal which professes to be devoted to nursing says in its issue of December 26th that the verdict of the jury remains upon the point of fact, and that it is quite certain that on any similar occasion in future similar proceedings will be taken, and that then the judgment would probably be upheld. We draw attention to this astonishing assertion so that nurses may see how much trust they may place in such self-constituted advisers. Where is the honesty of such a statement in view of the decision given on December 17th? The Master of the Rolls said: "The ground on which we refuse this motion is that there is no symptom of right in the people at such a meeting as this to have any question put at all." Mr. Justice Lopes said: "I agree with what Mr. Justice Wills said, that the idea is unprecedented, and that there was no right of any sort or kind which has been violated." Mr. Justice Rigby said, "I agree." The way in which the journal in question has suppressed all mention of this appeal and the decision upon it, and held out hope of success, if on any similar occasion similar proceedings should be taken, is most strange and misleading.

### SHORT ITEMS.

DESIGNS for a new Nurses' Home for Wandsworth Infirmary are being invited for competition, the cost not to exceed £10,000.—A "Member of the First and Parent Association in Bloomsbury Square" writes to the *Times* to express surprise that among the thirty-three elected members of the executive committee of "The Queen's Commemoration Fund" there is not one woman's name. —The second annual meeting of the Wallsend Nursing Association was held early in December, at which an encouraging report was presented. The association has met with most satisfactory support from the workmen of the local shipbuilding and engineering works.—Miss Broadwood recently gave an address on "Village Nursing" at a meeting of the Belvoir and District Benefit Nursing Association.—Lady William Beresford opened a successful bazaar in aid of the Dorking Nursing Association at the Oddfellows' Hall on December 16th. —The paper on "Nursing in Workhouses," read at the recent Poor Law Conference, was by an error in last week's issue attributed to Mrs. instead of to Mr. C. S. Roundell, a member of the Aged Poor Commission.

### Hygiene: For Nurses.

By JOHN GLAISTER, M.D., F.F.P.S.G., D.P.H.Camb., Professor of Forensic Medicine and Public Health, St. Mungo's College, Glasgow, &c.

### XXXVII. — DISINFECTION AND DISINFECTANTS (*Continued*).—HEAT AS A DISINFECTING AGENT.

In addition to chemical germicides, which act either by oxidation, or deoxidation, we have in heat a very valuable disinfector and bactericide, when applied either by dry air, moist air, or water. It is best adapted for the disinfection

FIG. 47.—WASHINGTON LYON'S PATENT. ALLIOTT AND PATONS' PATENT.

of personal clothing, bed-clothing, and room furnishings, such as carpets, rugs, cushions, &c., which have inadvertently been left in the infected room during the currency of the illness. For clothing which will not be injured, boiling in water for a space of twenty minutes, at least, is one of the best modes of disinfection. This is suitable for cotton and linen fabrics. For woollen fabrics, feathers, hair mattresses, &c., exposure to moist heated air, or steam, is the most suitable. This is usually done in a specially-constructed apparatus, which is commonly provided by the local sanitary authority. Dry heated air alone, or even under pressure, has been found by different experimenters in different countries to be ineffectual for this purpose, unless at such a temperature as would be likely to scorch the materials. Consensus of opinion, arrived at by direct experimentation, is now solely in favour of moist heat. In an efficient disinfector, where moist steam is used, the following are essential points, viz.: (1) That the steam under pressure should permeate every pore of the article to be disinfected; (2) that the temperature of the steam should be between 221° and 250° Fahr.; and (3) that the apparatus be so arranged that, alternately, hot dry air and moist steam, within the foregoing range of temperature, be passed into the chamber containing the articles to be disinfected. The machine, or disinfector, constructed on the Washington - Lyon principle by Messrs. Manlove, Alliott, and Co., of Nottingham, is one of the best, and a description of the mode of using it will suffice for this class of apparatus, of which there are several kinds with insignificant differences, and many makers. Fig. 47. The disinfector, which is oval in shape, and constructed of steel, consists of an outer and an inner chamber. The former is called the "jacket" of the latter, inasmuch as it envelopes the inner chamber. When the apparatus is working it is filled with steam at a pressure of 20 lbs. per square inch, and of a temperature of 267° Fahr. In the inner chamber is a movable cradle or basket, constructed of galvanised iron wire, in which the articles to be disinfected are placed. By means of suitable mechanical arrangements, communication may be established between the outer and inner chamber, both for filling the latter with steam and for emptying it partially of air. The disinfector, then, being in working order, let us follow from beginning to end of the process the mode of disinfecting, say, mattresses, pillows, &c. The inner chamber door is opened, the cradle is pulled out, and the articles are placed in it. It is again pushed back into the chamber, and the steam-tight doors are closed. Steam, under the conditions already mentioned, having been meanwhile circulating in the outer chamber, the air of the inner chamber is partially exhausted by the action of a steam nozzle, the effect being to create a partial vacuum in the inner chamber. At this stage the communication between outer and inner chamber is opened, and the steam rushes into the latter under considerable pressure, and forces itself into the interstices of the mattresses, &c. This part of the process lasts for not less than twenty minutes, the time being extended if the nature of the articles to be disinfected demand it. The communication between the two chambers is now closed, the steam in the inner chamber is now blown out, a partial vacuum is again established, and hot air takes the place of the steam. This hot air takes up any moisture which may have become condensed on the articles, which hereafter are taken out of the disinfector, the process of disinfection having been completed. Where large quantities of materials require to be disinfected daily, it becomes important that there should be no commingling of the infective with the disinfected articles. This is arranged by separating the inlet side of the machine from the outlet side (which are at opposite ends of the machine in this case) by a partitional wall, which is built at right angles to the length of the machine. Into the inlet opening the infective clothing is placed, which, after the process of disinfection is complete, is taken out by the outlet opening on

FIG. 48.—THRESH'S DISINFECTOR.

the other side of the wall. In this way, effectual separation of the infective from the disinfected material is ensured.

Another kind of disinfector, Fig. 48, based upon a different principle, has been recently invented by Dr. Thresh, Medical

Officer of Health for Essex, which is worthy of some attention, inasmuch as there are claimed for the machine efficiency, simplicity, and the non-requirement of skilled labour, coupled with safety. The principle upon which its construction is based is that water, containing a saline substance in solution, boils at a higher temperature, although at the same pressure, than pure water. The saline substance used in this machine is calcium chloride, and the machine is so contrived that the calcium salt originally put in may be used over and over again for a considerable time, water being gradually added to it by an automatic feed cistern. The temperature at which this saline solution boils is 225° Fahr., and the steam which is generated is made to circulate in a pipe which passes through this boiling solution. The machine itself, like the one already described, consists of an outer and inner chamber. The outer chamber contains the boiling saline solution and the pipe already mentioned. Into the inner chamber are placed the articles to be disinfected. When the temperature of this inner chamber reaches the temperature of the boiling solution, viz., 225° Fahr., the steam at the same temperature is made to enter, and thus it disinfects the materials. The time allowed for disinfection is practically the same as in the Washington-Lyon machine. When the disinfection is complete the course of the current of steam, by turning a lever, is now changed from the inner chamber into the flue of the machine, and thus, again, the inner chamber is converted into a dry-air chamber. Thereafter the disinfected goods are removed. The ingenuity of the inventor has been shown in the strength of the saline solution used, the boiling point of which is constant at 225° Fahr. This temperature, as has been pointed out by Dr. Barwise (who has experimented practically with the apparatus) in a paper in *Public Health*, "is not high enough to prevent condensation of the steam on the articles to be disinfected, and is sufficient to subsequently dry them." It is claimed for this machine by Dr. Barwise that it attains (1) uniformity of temperature, (2) rapidity of penetration, (3) maximum temperatures within the articles disinfected, and (4) dryness of articles disinfected. And the *Lancet* Special Sanitary Commissioners report of it that "the machine fulfils the conditions of a really efficient disinfector for all practical purposes" (*Lancet*, January 11th, 1896).

### Our Christmas Gifts for the Hospitals.

ALTHOUGH we offered no needlework prizes this year for competition, a number of our readers responded nobly to the appeal for contributions, and some nice bundles were sent last week to the following institutions: Charing Cross Hospital, the London Hospital, East End Mothers' Home, Clapham Maternity Hospital, North-Eastern Hospital for Children, Great Northern Central Hospital, North-West London Hospital, North London Hospital for Consumption, Hampstead, and the Royal Hospital, Richmond.

A constant contributor, Madame Monchablon, sent us seven boxes full of warm and useful things, notably, some delightful white flannelette bed jackets, with Turkey red collars, cuffs and pockets, dainty children's pink flannelette nightdresses, and fluffy woolly balls for the babies to play with, besides many other articles too numerous for mention. Welcome men's shirts (garments so much wanted, and so seldom sent, for they are not as attractive to make or so pretty to look at as frocks and petticoats) came from Nurse Clark, from Nurse Asbord, and from Madame Laczhovic; a boxful of beautifully-made petticoats and vests, knitted babies' bonnets, &c., from Nurse Ellen Clarke; petticoats and stockings from Miss Lockyer and Miss Willson; flannel petticoats and jacket from Miss Delap; knitted crossovers and comforters from "Aidyl"; and socks, petticoats, and other garments from a contributor who wishes to remain unknown. Besides these we received with much gratitude presents in money from Miss Newsom, Miss Annie George, Miss Kate Prior, and Miss Pritchard; to these funds we added enough to buy plenty of warm and useful vests, nightdresses, petticoats, and children's garments.

The parcels were very gladly received at their various destinations, though one could not but regret that they were not more bulky, and had contained a larger proportion of clothing for the men; for this there is always an unsatisfied demand at general hospitals. From Miss Elphick, lady superintendent of the North London Hospital for Consumption, and from the Governors of the Charing Cross Hospital we have received a kind letter of thanks, and Miss Blomfield, lady superintendent of the East-end Mothers' Home, writes: I beg to thank you very much indeed for your kind and generous gift of clothing for our poor mothers; we are always so grateful for any help of the kind.

### Queen Victoria's Jubilee Institute for Nurses.

ROLL OF QUEEN'S NURSES. JANUARY 1ST, 1897.

HER MAJESTY has been graciously pleased to approve of the appointment of the following as "Queen's Nurses":—

ENGLAND.—Superintendent: Eliza J. Rae, Liverpool. Nurses: Georgina Macleay, Elizabeth C. Jones, Mary E. Thomas, Margaret L. Costelloe, Maude E. Jacocks, Nellie O. Borham, Jane Horsley, and Maude B. Slater, London; Dora Mohun, Warwick; Jane Greig, Grimsby; Margaret G. Jones, Ruth Parker, Helena Lenton, and Charlotte Smith, Liverpool; Annie Lean, Droylsden; Aveline B. Mantell, Wisbech; Edith Milne, Kettering; Emily Forster and Margaret James, Brighton; Annie G. Forster, Eastbourne; Lily Haugh-Brown and Norah Cavenagh, Blackburn; Agnes M. C. A. White, Gateshead; Florence Appleby, Bingley; Jessie Ridley, Spalding; Winifred M. Pooler, Rawtenstall; Janet Blacklock and Ethel Dixon, York; Elizabeth A. Jones, Southampton; Martha M. Buxton, Leeds; Janet Rait Dall, Coventry; Margaret Jackson, Marion H. Purchase, Emily Tindle, Caroline A. Butler, Catherine M. Irwin, Rosa J. Horton, Kate O. Milligen, and Jane Burrow, Manchester; Gertrude Chadwick, Chorlton-cum-Hardy; Alice Price, Birmingham; Alice Marion Prior, Windsor.

WALES.—Nurses: Margaret A. Hodgson, Cardiff; Maggie Prytherick, Llandovery; Anne Turner, St. Bride's; Mary Warriner, Roch; Eliza A. Spencer, Llanbradach.

SCOTLAND.—Superintendent: Mary Jane Lamont, Glasgow. Nurses: Mary Hannah Bowlerwell and Hectorina Gillanders, Edinburgh; Mary Weir, Glasgow; Janet N. Borthwick and Annie Wishart, Aberdeen; Helen H. Anderson, Elgin: Jane Anderson, Mauchline; Catherine Mackinnon, Blantyre; Marion Sheridan, Inverness; Jean McIvor, Wick; Clara Burnett, Dalkeith; Isabella Jarron, Tobermory.

IRELAND.—Nurses: Sophia Pratt and Mabel W. Nunn, Dublin; Ellen M. Parsons, Enniskerry; Eleanor M. Moore, Dublin; Margaret E. Carden, Buncrana.

### LADY DUFFERIN'S FUND.

THE half-yearly examination of the pupil nurses at the Dufferin Hospital, Rangoon, Burma Branch, was held last month by Surgeon Lieut.-Colonel G. T. Thomas, when twelve candidates passed in midwifery and nursing.

### Legal Intelligence.

BREAY v. BROWNE.

In the Court of Appeal, December 17th. Present: The Master of the Rolls, Lord Justice Lopes, and Lord Justice Rigby.

Mr. Odgers, Q.C.: Will your Lordships allow me to make an *ex parte* application to you now?

The Master of the Rolls: Yes.

Mr. Odgers: I ask for leave to appeal from a decision of the Divisional Court given on Tuesday.

The Master of the Rolls: From the Divisional Court. Is leave required?

Mr. Odgers: Yes, my Lord. It was an appeal to them from the City of London Court.

Lord Justice Lopes: A county court case?

Mr. Odgers: Yes. The case went to the Divisional Court by way of appeal, and the Divisional Court set aside the verdict of the jury and entered judgment for the defendant, whereas judgment had been given for the plaintiff in the Court below. At the conclusion of the judgment I asked the Court for leave to appeal, and they refused me leave to appeal. I, therefore, come to your Lordships. My Lords, the action was of a somewhat unusual character. It was an action brought by a lady, Miss Breay, against the chairman of the annual general meeting of the Royal British Nurses' Association for refusing to put a resolution to the vote. The chairman refused to allow her to propose a resolution or to vote upon the resolution, of which she had given due notice. She gave three weeks beforehand notice by registered letter of the full text of her resolution. Her resolution appeared upon the agenda, but it was ruled out of order by the chairman on the erroneous ground that she had not sent notice of the resolution by registered letter. She at once stated that she had; she stated that she had paid a fee for the registered letter, and she handed up to him the certificate of registration, the piece of paper that is given to you when you register a letter, but, in spite of that, he ruled that it had not been sent by registered letter, he ruled it out of order, and he refused to allow her to propose it. It was a vote of censure on the committee; there was a protest signed by thirty-six matrons of the London hospitals as to the way in which the affairs of the Association had been managed; but it was ruled out, and she was prevented from bringing it forward. Miss Breay then brought her action in the City of London Court, and the question was left to the jury.

Lord Justice Lopes: What was the action for?

Mr. Odgers: The action was for damages for depriving her of her right.

Lord Justice Rigby: It is only nominal damages, is it not?

Mr. Odgers: She only recovered nominal damages—the Judge told the jury only to give nominal damages, and they only gave nominal damages, but he gave costs. It was submitted to the jury that he had acted partially from an indirect motive, and there was evidence to go to the jury, I submit, of partiality in the chairman, who acted from a desire to stop discussion at the moment.

The Master of the Rolls: I thought it was a verdict without a finding of malice.

Mr. Odgers: No, with malice. The Judge left to the jury, Did he act from an indirect motive? and they found that he did. The jury asked the question, "Did he see the certificate before he gave his decision?" And the Judge said, "No, he gave his decision before he saw the certificate, but the certificate was handed up to him, and he did not alter his decision." After he had seen that certificate of registration he persisted in ruling the resolution out of order.

The Master of the Rolls: It is immaterial.

Mr. Odgers: There was other evidence of malice. I may tell your Lordships that there had been a battle, I might call it, between the nurses and the doctors of this Association for some time, and the evidence of malice was that on a previous occasion a motion about a nurse had been ruled out of order by this chairman. The way the Judge left it to the jury was, Did he do it from an indirect motive, and the jury found that he did.

The Master of the Rolls: What was the indirect motive?

Mr. Odgers: The indirect motive was a desire to exclude this resolution, which was to be moved as a vote of censure on the Executive Committee, of which he was one.

The Master of the Rolls: On himself?

Mr. Odgers: On himself.

The Master of the Rolls: I cannot understand what right people have to have a motion put in that way.

Mr. Odgers: It is under the charter. This is a chartered association; and the plaintiff was one of the persons named on the charter to whom the charter was addressed.

Lord Justice Lopes: He was the chairman I suppose.

Mr. Odgers: Yes, my lord.

Lord Justice Lopes: I should have thought the duty of the chairman was to put it or not as he thought fit.

Mr. Odgers: He ruled it out on the ground that notice had not been sent in a registered letter.

The Master of the Rolls: He ruled it out.

Lord Justice Lopes: I think people are bound to do that. How can that give a cause of action, and in what shape? I cannot imagine.

Mr. Odgers: For violating her right, for depriving her of a right.

The Master of the Rolls: What right is there?

Mr. Odgers: The charter expressly says that every member shall have a right to vote at the annual general meeting.

Lord Justice Lopes: Supposing a member of Parliament rules a motion out, can they bring an action against him?

Mr. Odgers: All kinds of immunities are granted to members of Parliament, and even to learned judges.

Lord Justice Lopes: I thought it extended to every chairman.

Mr. Odgers: Here is a vote of censure on him and his colleagues.

The Master of the Rolls: Take the chairman of a railway company meeting. Supposing some gentleman wishes to move a vote of censure on the chairman, the directors, the staff, the railway guards, and the whole lot of them, and the chairman says, "What nonsense, I am not going to put this," do you suggest he could bring an action against him?

Mr. Odgers: That is not the way it was done. I cannot suggest that an action will lie for any honest mistake, but if a chairman rules out of order this motion of which due notice has been given.

The Master of the Rolls: He does not violate any right. There is no right.

Mr. Odgers: If your Lordship says so.

The Master of the Rolls: What law gives the right?

Mr. Odgers: The right, I submit, is this. If due notice of a motion is given in accordance with the bye laws under the charter, and it is there on the agenda, and you are there willing to bring the matter before the annual general meeting of the Association, then if it is excluded on a bye point such as saying it had not been sent in a registered letter when it had been so sent, and if that point is taken partially, with the object of getting rid of the motion, and with the object of getting rid of discussion, that, I submit, is violating the right, and an action lies on the principle of Ashby *v.* White and the other cases.

The MASTER OF THE ROLLS: There is no law which prevents us saying that we will not hear any more of this, or that we think it useless, and a waste of the time of the Court; there is no law to that effect; but you would say that an action lies against us because you have a right not to be stopped.

Mr. ODGERS: My lord, I think we have a right not to be stopped, but I do not think any action would lie against your Lordships for stopping us. I know that a judicial officer is clothed with immunity.

The MASTER OF THE ROLLS: I cannot conceive it in any case.

Mr. ODGERS: Take the case simply of a ministerial officer like a chairman.

The MASTER OF THE ROLLS: He is not a judicial officer.

Mr. ODGERS: He is not a Judge.

The MASTER OF THE ROLLS: What is he?

Mr. Justice LOPES: With regard to these parish councils I know a case which happened the other day where a man got up and wanted to put a resolution to the effect that our conduct with regard to the Armenians in Egypt was perfectly disgraceful. The chairman ruled it out of order. I know that myself. The chairman ruled that it could not be put.

Mr. ODGERS: I am sure no jury in that sense would find that there was indirect motive.

The MASTER OF THE ROLLS: And he did it for the purpose of smothering discussion.

Mr. ODGERS: This was a vote of censure on the committee. It was not suggested that the resolution was irrelevant, or was out of order, or anything of that kind, but the only ground for snuffing it out was that it had not been sent in a registered letter.

The MASTER OF THE ROLLS: You have just hit on the very phrase. The chairman thought it ought to be snuffed out, and he snuffed it out.

Mr. ODGERS: He snuffed it out on the ground that notice of it had not been sent in a registered letter when it ought, as he said, to have been sent in a registered letter; but it was sent in a registered letter, and Miss Breay handed up the certificate of registration to prove to him that it was sent by registered letter, yet the chairman insisted on ruling it out.

The MASTER OF THE ROLLS: There was no injury.

Mr. ODGERS: In Ashby *v.* White there was no injury, because the man for whom the vote was tendered got in. Your Lordship sees here was a resolution on the agenda paper, and it was ruled out on this highly technical ground, and I submit that there was as good a cause of action as there was in the case of Ashby *v.* White. In Ashby *v.* White a vote was tendered for the man who did get in; but it was held that that made no difference because the man had been refused his right to vote.

Lord Justice LOPES: Suppose a person is selected in whom everybody has confidence, and persons agree to abide by his decision with regard to any matter that arises, there s no right of action surely. If there is, I do not think people would care to be chairmen.

Mr. ODGERS: For an honest blunder I must admit that there would be no action; but here the jury had all the facts before them, saw the demeanour of the witnesses in the box, and they found that there was an indirect motive. The Divisional Court have set that finding aside.

Lord Justice LOPES: The only thing that astonishes me is that you were not nonsuited.

Mr. ODGERS: I was not in the Court below.

The MASTER OF THE ROLLS: You ought to have been nonsuited.

Mr. ODGERS: Your Lordship says so. One of the points taken in the Divisional Court was that there was no evidence of malice fit to be submitted to a jury, and Mr. Justice Wright took this view. He said: "The plaintiff here has probably sustained a wrong, but she has mistaken her remedy entirely. Her right, if any, was not a separate personal right, but a right as one of the corporators embraced in the company at large, and if she wanted a remedy, or if she now wants a remedy, her proper course was, or now is, for I do not see why she should not pursue it still, to bring an action in the name of herself and other shareholders, all or some, as the facts require, probably joining the company itself as plaintiff and bringing her action against the chairman and other directors asking for an injunction to restrain them from preventing the resolution being put to the meeting, and she might claim, in the alternative, a declaration that it ought to be put, and a claim in the nature, not of a prerogative mandamus, but a civil mandamus, to convene a fresh meeting and put the resolution. The plaintiff has not taken that procedure, therefore this action is entirely wrongly constituted. The sort of authority, one amongst many for the course she ought to have taken is the case of Pender *v.* Lushington in Chancery Division, page 70." When I applied to their lordships for leave to appeal Mr. Justice Wright said, "It seems to me quite plain that you have another remedy." I ventured to suggest that the time had gone by to apply for a mandamus, and then Mr. Justice Wright said, "not a prerogative mandamus; why should you not bring your action in the Chancery Division, or here? for an injunction to restrain them from putting the resolution, and for an order that there should be a fresh meeting at which it could be put?" That appears to be the ground of the decision of Mr. Justice Wright. Mr. Justice Wills did not put it on that ground, but Mr. Justice Wills put it upon the ground that the action was wholly without precedent, and that it appeared to him not to be founded upon any recognised principle, and neither in fact nor in law was there any ground for the action.

Lord Justice LOPES: I think I should have found as Mr. Justice Wills did.

Mr. ODGERS: This question applies to chairmen not only of chartered companies, limited liability companies, or, as your lordship suggested, parish councils, but it raises chairmen to the level of your lordships and extends the principle which has already been laid down.

The MASTER OF THE ROLLS: It would apply to a hole-in-the-corner meeting.

Mr. ODGERS: It would.

The MASTER OF THE ROLLS: You say it would apply to this: if a question were raised as to what you call the Armenian atrocities in a public house over their beer, and the gentleman at the top of the table, when he had put the beer to his mouth and drank a certain quantity, said, "I think this is all nonsense, I shall not put it."

Lord Justice LOPES: I am not sure that it would not apply to the man in the chair in the park when he puts a resolution.

Mr. ODGERS: It must be malicious.

The MASTER OF THE ROLLS: I should tell him, like a policeman, to move on. The ground on which we refuse this motion is that there is no symptom of right in the people at such a meeting as this to have any question put at all.

Lord Justice LOPES: I agree with what Mr. Justice Wills said that the idea is unprecedented, and that there was no right of any sort or kind which has been violated.

Lord Justice RIGBY: I agree.

### A PRESENTATION.

THE staff of the Blackheath and Richmond Institution have presented Mrs. Smiles and Miss Duncan with a very handsome set of dessert knives and forks as a Christmas gift.

### Everybody's Opinion.

[Correspondence on all subjects is invited, but we cannot in any way be responsible for the opinions expressed by our correspondents. No communication can be entertained if the name and address of the correspondent is not given, or unless one side of the paper only is written on.]

MRS. CHEADLE, Clapham, Brixton, and Surrey Institution of Trained Nurses, 210, Clapham Road, S.W., writes: I think it is right to tell you that there is a woman going about obtaining money under false pretences. She pretends to want to send a patient into an invalid home, and tells people she has lost her purse. On this pretext she got 5s. out of us, and I think it well to warn the public to be on their guard.

### A HOSPITAL WITHOUT MUSIC.

MISS M. ISLIP, Sister Matron the Royal Eye Hospital, Southwark, S.E., writes: Will you kindly, in your next issue of "THE HOSPITAL" NURSING MIRROR, correct a statement of "A Hospital Visitor," in a recent number, that "Gifts for the children and other patients from a kind friend have been forbidden at Christmas in this hospital." "A Hospital Visitor" has made a mistake; gifts would be most thankfully received by me for the patients, but we are not allowed to accept the offered Christmas tree for fear of fire.

### BED-MAKING.

A COUNTRY MEDICAL OFFICER writes: I would like your opinion, or that of some of your readers, on the following point in bed-making. My teaching is that there should be no blanket between the under sheet and the mattress or mackintosh when one is required. Our Government inspectors objected to finding a couple of beds in the lunacy department without an under blanket. From what I understand this seems to be the opinion of both lunacy and Government inspectors, lay or medical. From a perusal of numerous nursing text-books opinion seems divided, my own idea being that if a patient is properly attended to and lying on a properly made mattress such blanket only tends to produce bedsores. Miss Nightingale is very explicit on that point.

### POST-PARTUM HÆMORRHAGE.

REFERRING to the midwifery article on "Post-Partum Hæmorrhage" which appeared in "THE HOSPITAL" NURSING MIRROR on November 28th, Dr. G. de G. Griffith writes as follows concerning the advantages of compressing the aorta, which he describes as the very best method of arresting the bleeding. He says: I used always to be extremely anxious when such cases occurred, or when I was called to them occurring in the hands of others. But having for years practised only this method of treatment in even the very worst cases, and having never, even in one instance, had it fail, I now never think of adopting any other plan. It may be practised at once on the very first appearance of loss at the same time that the uterus is compressed. I have never known it fail, even in the very worst instances, and, as a rule, the hæmorrhage is immediately checked, and quite stopped in fifteen or twenty minutes; nor have I ever after the lapse of half an hour known it to return, though continuous compression had not been kept up that length of time.

### Appointments.

### MATRONS.

OPHTHALMIC SCHOOLS, HANWELL.—Miss E. Lynch has been appointed Superintendent of Nurses at the above institution. She was trained at St. Bartholomew's Hospital, Rochester, where she has since had the charge of various wards.

ROTUNDA HOSPITAL, DUBLIN.—Miss Lucy Ramsden has been appointed to the post of Superintendent at this hospital. Miss Ramsden is an Englishwoman, from Yorkshire, and was trained at St. Thomas's Hospital, London, afterwards working for a year at the Manchester Fever Hospital, from whence she went to the Rotunda as head staff nurse of the Surgical Wing. This responsible position she has filled for five years. We congratulate Miss Ramsden on her promotion.

### Christmas at the Hospitals.

### ST. THOMAS'S HOSPITAL.

At St. Thomas's Hospital the patients had their usual Christmas fare, and in the afternoon each was permitted to have one friend to take tea. The nurses who enjoy the pleasant quarters which the new nursing home affords, shared their advantages with their companions by inviting them to an afternoon "At Home," which was voted a great success. On Boxing Day the Matron (Miss Gordon) entertained a large company of past and present Nightingale nurses at tea, after which there was carol singing in the wards. Miss Haig-Brown (the home sister) conducted, and a number of pretty carols were rendered with much spirit. The wards were charmingly decorated and illuminated, the delicate hues of the coloured lights having also a beautiful effect from without. Many passers-by stopped on Westminster Bridge to look at the festive glow which appeared in the numerous windows, penetrating even the dulness of a December evening on the Thames.

### THE LONDON HOSPITAL.

On Christmas morning the patients at the London Hospital began the day with carols, sung as usual by a long procession of nurses who made the tour of the wards. A delightful Father Christmas was the next visitor, and he bestowed gifts and kindly greetings on all sides. After dinner the patients' friends were admitted to visit them for an hour, and later on sumptuous teas were served. The decorations of the wards and the prettily-spread table gave great pleasure and elicited much admiration from those for whose benefit this seasonable display is always made. The children's wards were centres of attraction and never looked more charming than on the present occasion. The students, who of late years have entirely relieved the nursing staff of all trouble with regard to the entertainment department, surpassed themselves this Christmas, and may well be congratulated on their admirable management. A dress rehearsal on the previous evening had enabled Miss Lückes, the matron, and many nurses to enjoy the very clever items which made up the programme eventually carried out in the wards. So well and punctually did the amateur actors play their parts, that piece succeeded piece, and the patients had no "long waits" between farce, waxworks, concert, the Pierrot Troupe, and variety entertainments. Precisely at half-past eight the strains of "God Save the Queen" were heard in every ward, and when the house-governor made his official round of inspection quiet reigned, and patients were settled down peacefully to sleep after the happiest festival that had ever fallen to the lot of the majority of these East-end men and women.

### MIDDLESEX HOSPITAL.

The usual Christmas festivities have been going on during the past week at Middlesex. Plenty of pleasant fare in the shape of turkeys, geese, and plum puddings was shared on Christmas Day by all who could enjoy these "delicacies of the season"; from a huge tree erected in the board-room useful gifts were distributed to all well enough to be present while for those unable to leave the wards was provided the excitement of a bran pie. The wards were prettily decorated, and the patients were allowed visits from their friends during the afternoon. On New Year's Day takes place the children's treat, Christmas trees in each ward, that one in Princess May ward being lighted by electricity.

### Minor Appointments.

CHUNG-KING, CHINA. — Miss Hannah Rosher has been appointed Nursing Sister at this station. She was trained at the London Hospital, afterwards for four years working o the private staff of that hospital.

### For Reading to the Sick.

### THE NEW YEAR.

### Verses.

ONCE to every man and nation comes the moment to decide
In the strife of Truth with Falsehood for the good or evil side!
Some great cause, God's new Messiah, offering each the bloom or blight,
Parts the goats upon the left hand, and the sheep upon the right,
And the choice goes by for ever 'twixt that darkness and that light.—*Lowell.*

Man must pass from old to new,
From vain to real, from mistake to fact,
From what once seemed good, to what now proves best;
How could man have progression otherwise.—*Browning.*

This is the day which Thou hast made,
Almighty Father! Thou,
Who once the earth's foundations laid,
Fashion'd its every hill, and glade,
And valley, green and low.
Shall we not then all gladly rise,
Rejoicing every morn?
Lifting to Thee our trustful eyes;
Knowing each moment as it flies
But nears a brighter dawn.—*Mrs. Howard.*

The Year begins with Thee,
And thou begin'st with woe,
So let the world of Sinners see
That blood for sin must flow.—*Keble.*

Then be it so!
For in better things we yet may grow,
Onward and upward still our way,
With the joy of progress from day to day;
Nearer and nearer every year
To the visions and hopes most true and dear!
Children still of a Father's love,
Children still of a Home above!
Thus we look back
Without a sigh o'er the lengthening track.
—*F. R. Havergal.*

### Reading.

This is the day which the Lord hath made, we will rejoice and be glad in it.—*Ps.* cxviii. 24.

So teach us to number our days: that we may apply our hearts unto wisdom.—*Ps.* xc. 12.

I am Alpha and Omega, the Beginning and the End, the First and the Last.—*Revelation* xxii. 13.

O Lord, Thou knowest what is best for us; give what Thou wilt, and how much Thou wilt, and when Thou wilt! Deal with us as Thou thinkest good, and as best pleaseth Thee, and is most to Thine honour. Set me where Thou wilt, and deal with me in all things just as Thou wilt!

God is our Last End as well as our First Cause. God possessed, our own God, that is creation's home, our last end, there only is our rest. Another day is gone, another week is passed, another year is told. Blessed be God, then, we are nearer to the end. It comes swiftly, it comes slowly too. Come it must, and then it will all be but a dream to look back upon. But there are stern things to pass through, and to the getting well through them there goes more than we can say. One thing we know, that personal love of God is the only thing which reaches God at last.—*F. W. Faber.*

### Where to Go.

ST. MARY'S DAY NURSERY AND HOSPITAL FOR SICK CHILDREN, PLAISTOW.—Children's entertainment on Saturday, January 2nd, three to six o'clock.

ST. MARY'S HOSPITAL, W.—Patients' Christmas entertainment, Wednesday, January 6th, half-past seven p.m.

METROPOLITAN HOSPITAL, KINGSLAND ROAD.—Entertainment for the patients, Thursday and Friday, January 7th and 8th, four to half-past seven p.m.

CRITERION THEATRE.—A matinée of "Liberty Hall," in aid of the funds of the Royal British Nurses' Association, will be given on Thursday, January 14th, by permission of Mr. Charles Wyndham, at three o'clock. The performance is under Princess Christian's patronage, and the Duchesses of Sutherland and Buccleuch, the Marchioness of Londonderry the Countess of Clanwilliam, Lady Jeune, and others have also given their names as patrons. Tickets may be obtained from Miss L. Caledon-Alexander, 137, Victoria Street, W.; the Hon. Mrs. Bevan, 4, Lower Berkeley Street, W.; Miss Beaumont, Buckland Court, Betchworth; or Lady Duckworth, 11, Grafton Street, Piccadilly.

ROYAL BRITISH NURSES' ASSOCIATION.—The secretary requests us to state that the quarterly meeting of the General Council will be held on Friday, January 8th, at five p.m. The course of demonstrations on invalid cookery will be resumed at the offices of the Association, 17, Old Cavendish Street, on Tuesday, January 12th, at half-past two p.m., and will be continued on the 19th and 26th inst., the syllabus for each lesson being as follows: Eighth lesson—Oyster soufflée, stewed tripe, stewed mushrooms, apple jelly, tapioca cream, coffee. Ninth lesson—Mutton broth, boiled mutton, parsley sauce, rusks, blancmange, œufs à madame, barley water. Tenth lesson—Clear soup, savoury custard, arrowroot soufflée, boiled whiting, rissoles, wine whey.

### Notes and Queries.

*District Nursing.*

(75) I am thinking of starting a district nurse in a country village, and should be glad of information as to the probable expenses involved, and the usual arrangements and payment?—*E. H. M.*

Write to the Secretary, Queen Victoria Jubilee Institute for Nurses, St. Katharine's Hospital, Regent's Park, N.W., and ask for the information you require.

*Massage.*

(76) I want to learn massage and gain a certificate for it. How can I learn in a country town?—*A.M.S.*

You should obtain the certificate of the Society of Trained Masseuses. Write to the hon. Secretary, 12, Buckingham Street, Strand, W.C., enclosing a stamped envelope for reply.

*Nursing at Rio.*

(77) I want to get to Rio to the Strangers' Hospital. Can you tell me if there is a vacancy for a sister?—*Sister.*

Refer to "THE HOSPITAL" NURSING MIRROR for Aug. 8th, 1896, p. clviii, and Oct. 10th, 1896, p. 14. If you wish to know about vacancies you can only apply to the Matron direct.

*A Book for Mothers.*

(78) Nurse F. would like to know of a good and useful medical book for a mother, far from any doctor?

"The Mother's Help and Guide to the Domestic Management of Children," by P. Murray Braidwood, M.D., published by the Scientific Press, 28 and 29, Southampton Street, Strand, W.C.

*Dispensing.*

(79) I am very anxious to learn dispensing. Can you tell me if there are any classes in Edinburgh which women could attend, or could I be coached by correspondence for the Apothecaries' Hall examination?—*Edinburgh.*

Write to Miss Bradbury, Resident Dispenser, at the Ryde Dispensary, Isle of Wight. We feel sure she will be pleased to answer your questions and to help you, if she can, with advice.

*Private Nursing Institutions.*

(80) A correspondent asks if it is legal for a private nursing institution to include in its rules one which prohibits nurses joining it from leaving to take up private work on their own account in the neighbourhood, under a pecuniary penalty. Copy of rules enclosed.

An institution may make what rules it likes in this way, which will be binding upon nurses signing an agreement to abide by them. The rules of the institution in question certainly show that nurses will best consult their own interests by not joining one managed on the principle of getting as much as possible out of the nurses for inadequate pay, while the profits on their earnings go to swell the private purse of the proprietors.

## Figures and Tables

**Fig. 47. f1:**
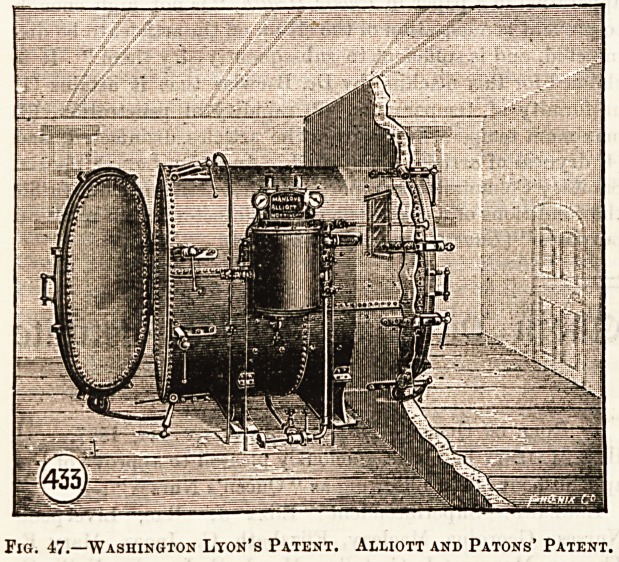


**Fig. 48. f2:**